# Spontaneous Hemarthrosis and Compartment Syndrome in an Elderly Female With Acquired Hemophilia A: A Case Report

**DOI:** 10.7759/cureus.95170

**Published:** 2025-10-22

**Authors:** Mohammed S Alam, Mahfuza Khan, Roxana Lazarescu

**Affiliations:** 1 Medicine and Surgery, Wyckoff Heights Medical Center, New York, USA; 2 Surgery, Wyckoff Heights Medical Center, New York, USA; 3 Internal Medicine, Wyckoff Heights Medical Center, New York, USA

**Keywords:** acquired hemophilia a, compartment syndrome, elderly patient, factor viii inhibitor, intramuscular hematoma, spontaneous hemarthrosis

## Abstract

Acquired hemophilia A (AHA) is a rare but life-threatening bleeding disorder caused by autoantibodies against factor VIII. Unlike congenital hemophilia, AHA often presents in older adults without a prior bleeding history, manifesting with spontaneous, severe bleeding into soft tissues, muscles, or joints. We report the case of a 79-year-old woman with osteoporosis and osteoarthritis who presented with progressive right elbow pain and swelling, initially suspected to be septic arthritis or synovial tumor. Her course was complicated by massive hemarthrosis, intramuscular hematoma, and compartment syndrome requiring fasciotomy. Laboratory evaluation revealed a prolonged activated partial thromboplastin time (aPTT), markedly reduced factor VIII activity, and the presence of inhibitors, confirming the diagnosis of AHA. The patient was successfully managed with prothrombin complex concentrate, corticosteroids, and surgical intervention. This case highlights the importance of early recognition of AHA in patients with unexplained bleeding and prolonged aPTT, as misdiagnosis can delay life-saving treatment.

## Introduction

Acquired hemophilia A (AHA) is a rare autoimmune disorder with an estimated incidence of 1-1.5 cases per million annually [1]. Most patients are elderly, and bleeding often presents spontaneously without trauma or a family history of bleeding disorders [2]. Delay in recognition increases morbidity and mortality due to uncontrolled bleeding or complications such as compartment syndrome [3-5]. AHA results from the development of inhibitory autoantibodies against clotting factor VIII, leading to impaired coagulation [6]. Misdiagnosis is common because clinical presentation can mimic septic arthritis, malignancy, or traumatic injury [7]. Management of AHA involves both hemostatic control and eradication of the inhibitor [8]. Here, we present a case illustrating this diagnostic challenge and its potentially limb-threatening consequences.

## Case presentation

A 79-year-old woman with a history of osteoporosis and osteoarthritis presented to the emergency department with a two-week history of progressive pain and swelling in her right elbow. On arrival, her elbow was visibly swollen, warm, and tender, with marked limitation of movement. The swelling extended into her hand, which showed discoloration. The initial clinical impression raised concern for septic arthritis or a possible synovial tumor. Pain was described as progressive but not associated with systemic symptoms, which contrasts with septic arthritis (acute, severe pain with fever and elevated inflammatory markers) or malignancy (often chronic swelling with systemic features).

Laboratory investigations (Table 1) quickly shifted the diagnostic focus. Her activated partial thromboplastin time (aPTT) was markedly prolonged and failed to correct with a mixing study. Factor VIII activity was reduced to 2%, and a lupus anticoagulant was detected. In addition, her hemoglobin had fallen to 7 g/dL, and creatine kinase was elevated to over 2,000 U/L. Viral serology and lymphoma panel were negative.

**Table 1:** Summary of investigations. PT: prothrombin time; INR: international normalized ratio; aPTT: activated partial thrombplastin time; CPK: creatinine phosphokinase

Parameters	Patient values	Reference range
Hemoglobin	7 g/dL	12–16 g/dL
Hematocrit	22%	36–46%
Platelet count	154 × 10⁹/L	150–400 × 10⁹/L
aPTT	98.6 seconds	25–35 seconds
PT/INR	Normal	PT: 11–14 seconds; INR: 0.8–1.2
Factor VIII activity	2%	70–180%
Factor VIII inhibitor	Positive	Negative
Mixing study	No correction	Correction expected in deficiency
Lupus anticoagulant	Positive	Negative
CPK	2,012 U/L	26–192 U/L
Iron studies	Normal	Normal
Haptoglobin	305 mg/dL	34–200 mg/dL
Hepatitis A, B, C serologies	Negative	Negative
Acute lymphoma panel	Negative	Negative

**Figure 1:**
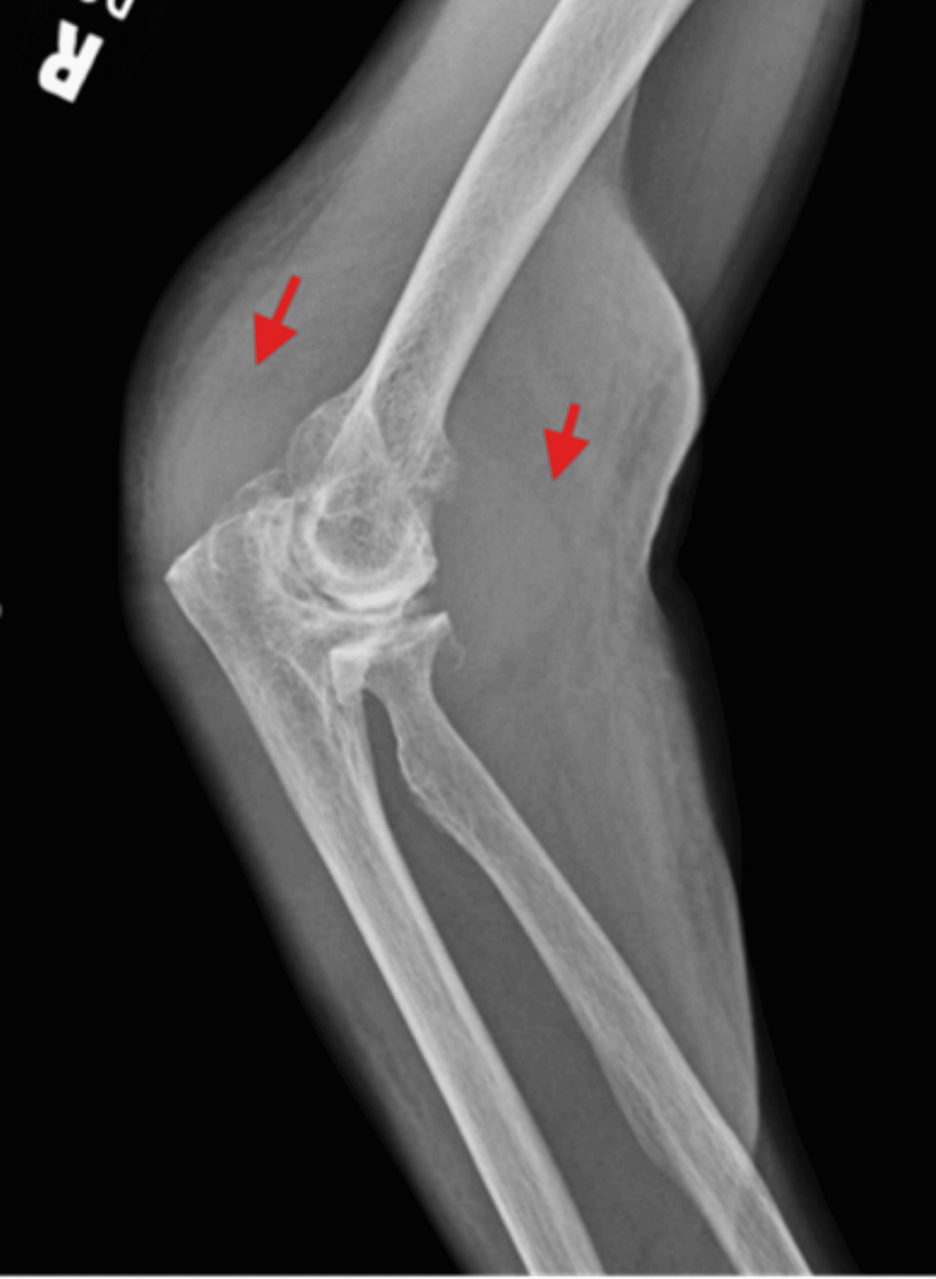
X-ray of the right elbow showing large right elbow joint effusion with soft tissue swelling (red arrows).

**Figure 2:**
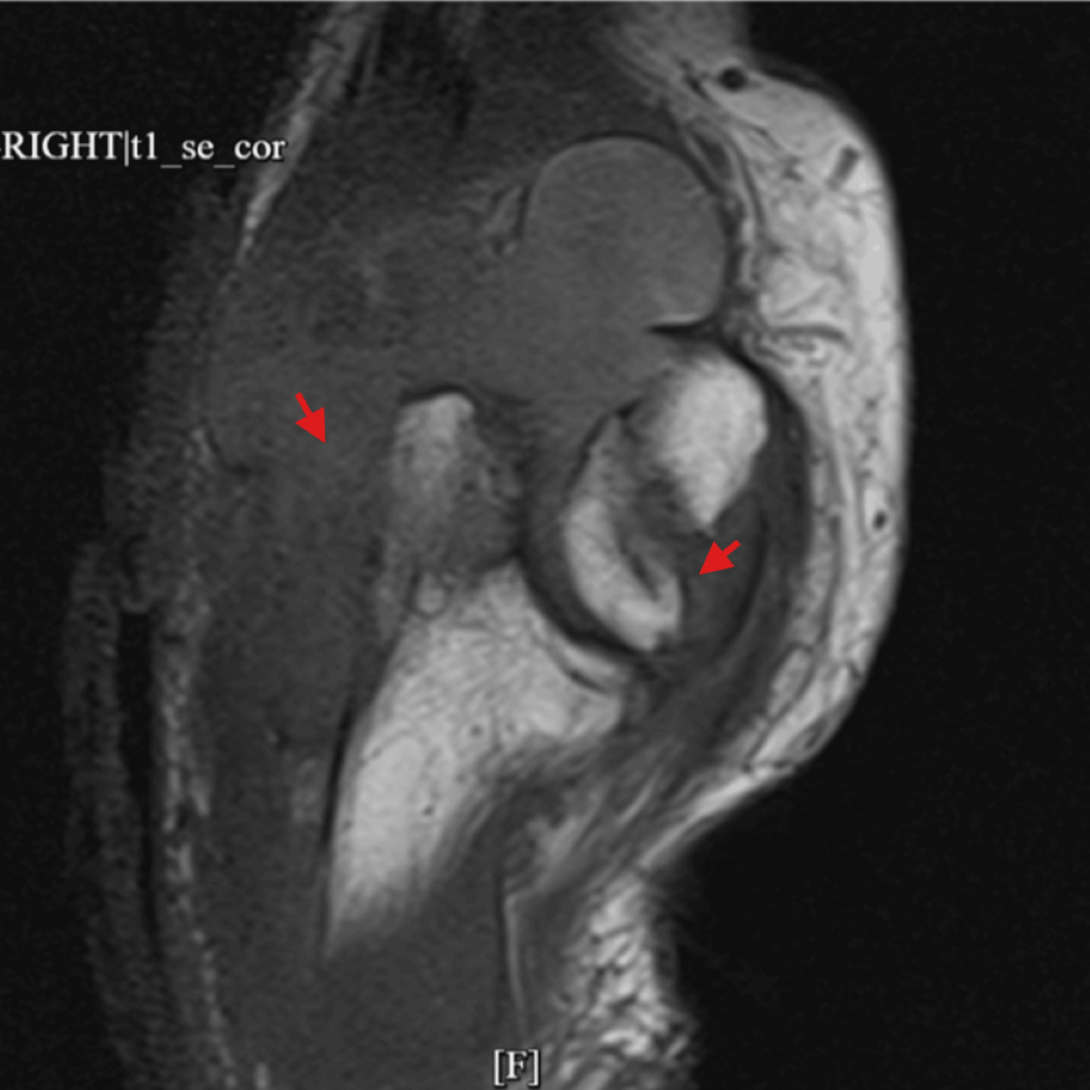
MRI of the right elbow with and without contrast showing large, complex, heterogeneous, partially enhancing fluid and synovium within the elbow joint anteriorly and posteriorly (red arrows).

**Figure 3:**
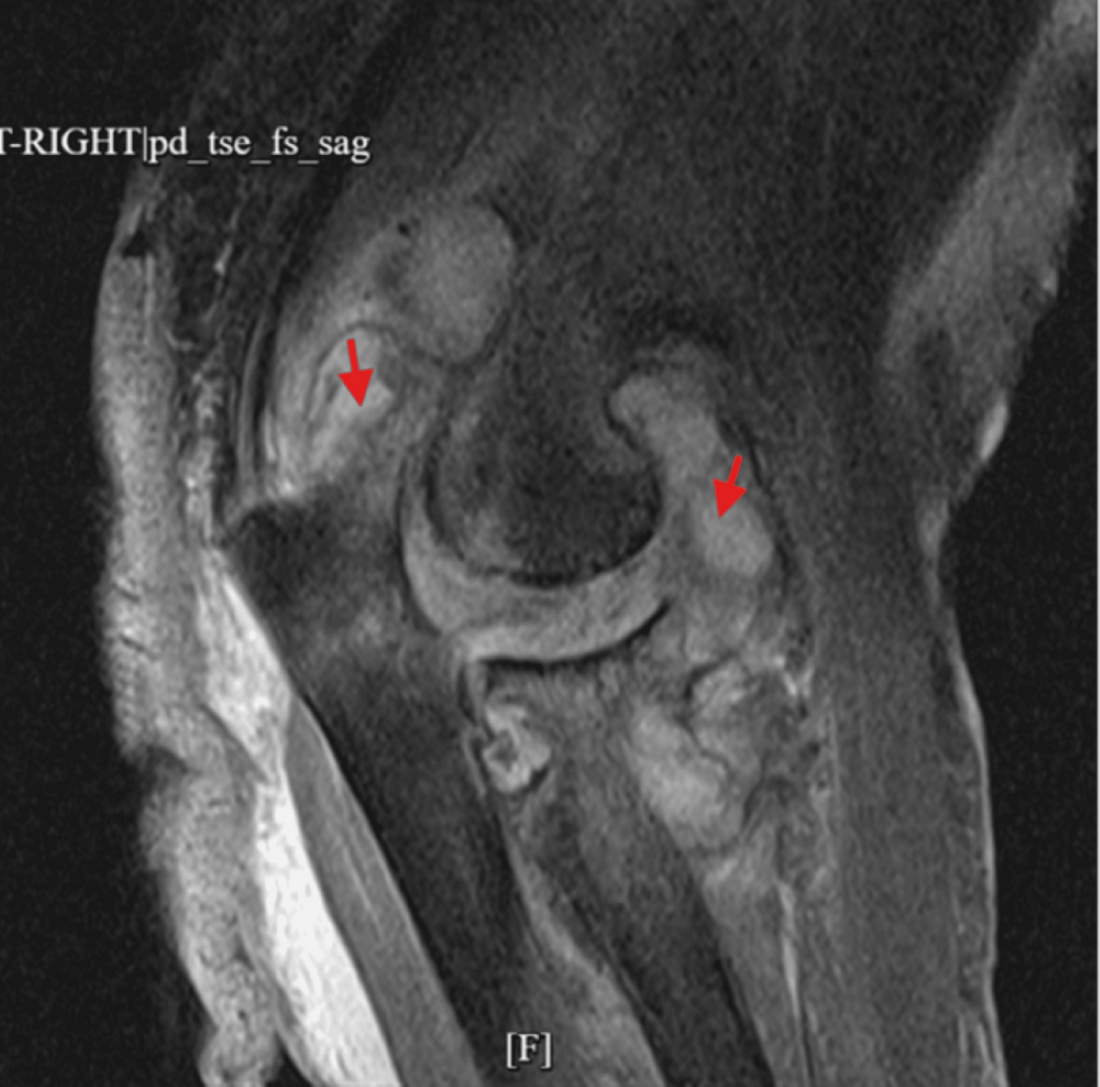
MRI of the right elbow with and without contrast showing large, complex, heterogeneous, partially enhancing fluid and synovium within the elbow joint anteriorly and posteriorly (red arrows).

Imaging supported a significant joint and soft tissue process. X-ray showed a large elbow effusion with soft tissue swelling (Figure 1). MRI revealed a large heterogeneous fluid collection with enhancing synovium, marrow edema, and intramuscular edema (Figures 2, 3). Ultrasound suggested a nonspecific intra- or extra-articular lesion.

A synovial biopsy was performed, but it only showed nonspecific changes without malignancy. Markedly prolonged aPTT uncorrected by mixing studies, severely reduced factor VIII activity, and a confirmed lupus anticoagulant established the diagnosis of AHA.

Her clinical course worsened as she developed compartment syndrome of the right upper extremity, requiring urgent fasciotomy and evacuation of a hematoma. Hematology confirmed the diagnosis of AHA, and treatment was started with prothrombin complex concentrate at 50 U/kg (3,000 units total) and prednisone 50 mg daily, with pantoprazole added for gastric protection. Rituximab was planned but deferred while awaiting family consent. Because of the anticipated prolonged course of corticosteroids, Bactrim prophylaxis was started to reduce the risk of *Pneumocystis* infection.

**Figure 4:**
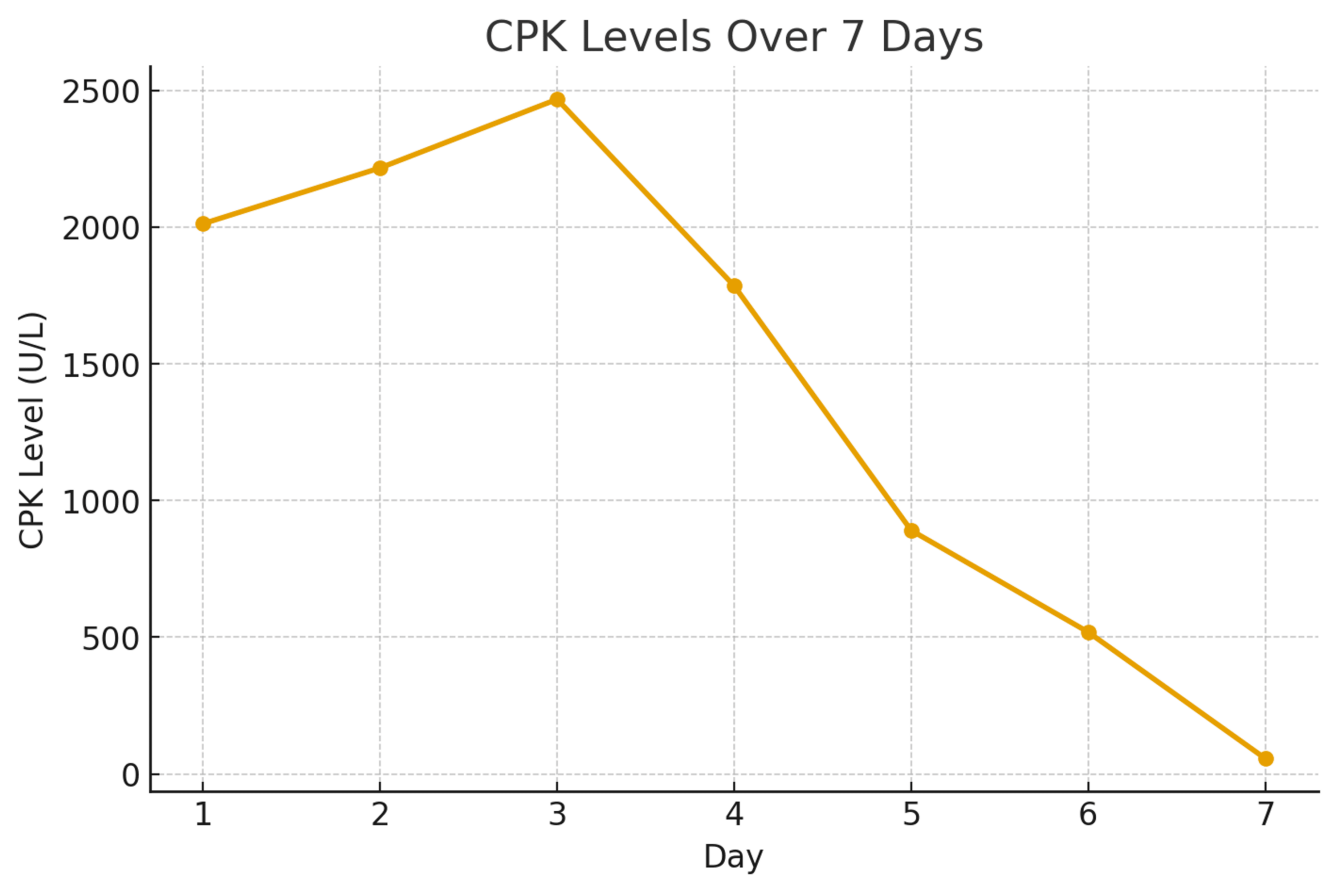
Graph showing CPK levels steadily decreasing over time. X-axis: hospital days. Y-axis: CPK in U/L. CPK: creatinine phosphokinase

**Figure 5:**
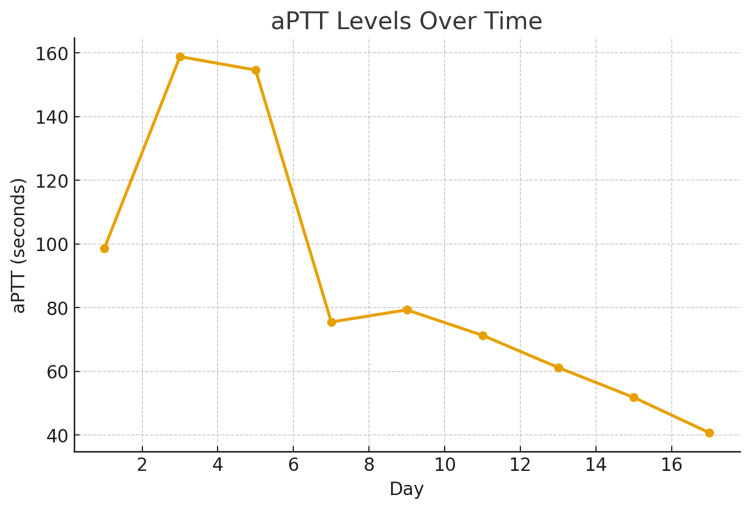
Graph showing gradual declining of aPTT level over time. X-axis: hospital day. Y-axis: aPTT in seconds. aPTT: activated partial thrombplastin time

Over the following days, her bleeding stabilized with prothrombin complex concentrate (PCC) and prednisone. Her creatinine phosphokinase and aPTT gradually improved, though aPTT remained prolonged because of the coexisting lupus anticoagulant (Figures 4, 5). Her limb function improved after the fasciotomy, and she was able to begin rehabilitation. She was discharged home on prednisone with instructions for close hematology follow-up and weekly monitoring of factor VIII levels and inhibitor titers.

## Discussion

AHA is a rare bleeding disorder caused by inhibitory autoantibodies against factor VIII. It typically affects the elderly and presents with spontaneous bleeding in soft tissues or mucous membranes, although hemarthrosis is less common than in congenital hemophilia [1,6,8]. While hemarthrosis is more commonly associated with weight-bearing joints such as the knee, it can also affect non-weight-bearing joints such as the elbow [9].

This case underscores that early recognition of AHA requires careful attention to subtle distinguishing features. Key clues include acute or progressive joint swelling without trauma, absence of systemic symptoms such as fever, and disproportionate bleeding relative to the apparent severity of joint findings. In this case, the patient presented with symptoms mimicking septic arthritis or a synovial tumor, initially delaying diagnosis. Septic arthritis was excluded based on synovial fluid analysis, which demonstrated bloody aspirate without evidence of infection, and negative cultures for bacterial growth [7]. Synovial tumor, including pigmented villonodular synovitis, was excluded on histopathological examination, which demonstrated hemorrhagic and fibrotic tissue without neoplastic features [7]. Hemarthrosis secondary to congenital or systemic coagulopathies was excluded, as the patient had no prior bleeding history and laboratory studies (prothrombin time, fibrinogen, platelets, liver function tests) were normal, making congenital hemophilia, von Willebrand disease, or consumptive coagulopathy unlikely [6]. This systematic exclusion of alternative causes supported the diagnosis of AHA, characterized by an isolated prolonged aPTT, reduced factor VIII activity, and the presence of factor VIII inhibitors [3,4]. Compartment syndrome due to intramuscular hematoma is an unusual but serious complication of AHA [3-5]. Prompt surgical decompression alongside hemostatic therapy is required to preserve limb function. Management of AHA involves both hemostatic control and eradication of the inhibitor. Bypassing agents (recombinant activated factor VII, activated PCC) or, less commonly, PCC can be used for bleeding control [7]. Immunosuppression with corticosteroids, rituximab, or cyclophosphamide is the mainstay of inhibitor eradication [2,8].

This case emphasizes the importance of considering AHA in elderly patients with unexplained bleeding and isolated prolonged aPTT, as timely hematology input can be life-saving.

## Conclusions

AHA should be suspected in elderly patients with unexplained bleeding and prolonged aPTT uncorrected by a mixing study. Hemarthrosis in AHA is rare but may mimic septic arthritis or synovial tumors. Compartment syndrome from intramuscular hematoma is a rare complication requiring urgent surgical intervention. Optimal management requires both hemostatic therapy (PCC, recombinant activated factor VII). Awareness of this rare presentation is crucial for avoiding misdiagnosis and initiating timely, life-saving therapy. This report highlights that prompt suspicion based on atypical presentations and prolonged aPTT can prevent diagnostic delays and improve short-term and long-term outcomes.
